# Prevalence of Depression in Retirees: A Meta-Analysis

**DOI:** 10.3390/healthcare8030321

**Published:** 2020-09-04

**Authors:** Manuel Pabón-Carrasco, Lucia Ramirez-Baena, Raúl López Sánchez, Isabel Rodríguez-Gallego, Nora Suleiman-Martos, José L. Gómez-Urquiza

**Affiliations:** 1 Spanish Red Cross Nursing School, Sevilla University, 41009 Sevilla, Spain; mpabon@cruzroja.es (M.P.-C.); isroga@cruzroja.es (I.R.-G.); 2 Internal Medicine, Hospital Quirónsalud Barcelona, 08023 Barcelona, Spain; aullopezsanchez92@gmail.com; 3 Nursing Department, Faculty of Health Sciences, University of Granada, Campus University of Ceuta, 51001 Ceuta, Spain; norasm@ugr.es; 4 Faculty of Health Sciences, University of Granada, 6018016 Granada, Spain; jlgurquiza@ugr.es

**Keywords:** aging, depression, retirement, health personnel, nursing

## Abstract

*Background:* Retirement is a final life stage characterized by the ceasing of work and the loss of a routine, social relations, role, status, accomplishments, and aspirations, etc. Many times it is accompanied by negative feelings and can provoke different psychoemotional reactions such as depression, among others. The aim of this study is to analyze the prevalence of depression, as well as its psychoeducational approach in retirees. *Methods:* A paired systematic review with meta-analysis was conducted in different databases—Medline, Scopus, CUIDEN, CINAHL, LILACS and PsycINFO. Original studies were included in English, Spanish and French that were published in the last 10 years, and which approached depression in retirees. *Results:* A total of 11 articles were selected after applying inclusion and exclusion criteria. The mean value of the prevalence levels of depression in retirees obtained in the meta-analysis was 28%. Depression is more frequent in retirees, with mandatory retirement, retirement due to illness, and anticipated retirement presenting higher levels of this disease. The health role in the psychoeducational approach is highlighted in 41.6% (*n* = 5). Conclusions: With almost one-third of retirees suffering from depression, it is necessary to implement prevention and early detection measures to approach a public health problem.

## 1. Introduction

According to the World Health Organization (WHO), depression is a mental disorder that manifests itself through sadness, extreme apathy, anhedonia, feelings of guilt, low self-esteem, sleep disorders, appetite, lack of concentration, and sensation of tiredness [1].

The WHO estimates that depression affects more than 300 million people in the world [2]. In its most serious form, this disease can lead to suicide, since 800,000 individuals commit suicide each year in the world, this being the second most common cause of death in individuals between 15 and 29 years old [2].

Even so, depression affects all age ranges, especially vulnerable individuals such as retirees. Retirement is a transition which occurs in the last stage of life and is characterized by the ceasing of work and, with that, the loss of a routine, social relations, role, status, accomplishments, and aspirations, etc. [3]. This implies changing the lifestyle adopted during many years in the working stage and supposes a phenomenon that can alter the psychosocial realm of the retiree [4]. Additionally, the aging process supposes diverse changes in health and it would lead to the decline of individuals who suffer it, altering their self-image, self-esteem, autonomy, and functionality [5].

The majority of individuals understand the transition from being active in working life to retirement as the process by which they start to become old [3], which generates feelings of uselessness, thus predisposing them to depression.

Depression is one of the most underdiagnosed diseases and, still in the 21^st^ century, it is still being stigmatized or even banalized, ignoring the resources, treatments, and recovery of this cruel disease [6].

Research on the depression of retirees is very scarce, and few studies directly relate depression with retirement [7], despite the great diversity of published articles on this disease [8]. Currently, the publications on depression are directed to valuing different treatments to determine if they are effective such as, for example, the use of acupuncture, music therapy, and the search for the ideal antidepressant [9,10,11].

Although the current research lines on the search for effective treatments against depression are important, more studies should be conducted on the direct relation between retirement and depression. It is also important to know the prevalence of depression and how health professionals, including nurses, can collaborate on a psychoeducational approach to lower the prevalence figures.

The fact is that, both in health prevention and promotion, nursing plays a very prominent role to stop the progress of this disease [12]. Nursing is very important in early detection [7], apart from its fundamental contribution to the psychoeducational approach [12]. These health professionals are also in charge of maintaining a close relationship with patients and their relatives, so as to foster a good use of pharmacological treatments, increasing adherence to them [13,14] and thus preventing future relapses [7].

Therefore, the main aim of this paper is two-fold: (a) to analyze the prevalence of depression in retirees; (b) to analyze the role of the psychoeducational approach to depression.

## 2. Methodology

### 2.1. Data Sources and Inclusion Criteria

A systematic review with meta-analysis was conducted until March 2020, following the recommendations of the Preferred Reporting Items for Systematic Reviews and Meta-analyses (PRISMA) statement [15]. The following scientific databases were consulted: Medline, Scopus, CUIDEN, CINAHL, LILACS and PsycINFO.

The inclusion criteria for the selection of studies were: original studies in English, Spanish and French; time restriction to the last 10 years; a theme related to retirement and depression diagnosed by healthcare professionals with the prevalence confirmed by a validated scale, including all the modalities of retirement (anticipated, preretirement, due to disease, voluntary or mandatory) with no age limit in retirement. The last 10 years were marked to appreciate the effect of the global economic crisis in 2008. Many people had to advance their retirement age and it has been a period of interest at the social, health and economic level. The exclusion criteria were studies not available in full text, a mandatory retirement whose cause was depression and articles of low methodological quality, as well as reviews, clinical cases or expert opinions.

### 2.2. Search Strategy

The search equation was elaborated by means of terms (MeSH) used to identify the primary studies: retirement, depression, aged, nursing and “health Personnel”.

Table 1 shows the different search equations conducted in each database, with the filters used and the results obtained in each of them.

### 2.3. Study Selection

This manuscript is registered in PROSPERO with the following reference code: CRD42020163979. A preselection was conducted by pairs, where the results were screened based on the inclusion criteria. Subsequently, the full text was read and then a critical reading was conducted to evaluate the validity of the study following the recommendations of Cooper, Hedges and Valentine [16].

For each selected study, a back-and-forth search was performed to locate more research studies related to the theme of interest. In case of a disagreement between the two team members, a third researcher was consulted, blinded to his peers’ decision and who followed the same search criterion protocol [17]. Finally, a critical reading was performed of the chosen articles to evaluate possible biases in the methodology [16]. Apart from that, a search was performed by an expert in the grey literature to identify potentially valid articles.

Starting from an initial search of 556 articles, 179 were selected after eliminating duplicates using the Mendeley^®^ bibliographical manager and applying the inclusion criteria. After reading titles and abstracts, 148 articles were deleted, with a total of 31 articles remaining.

**Figure 1:**
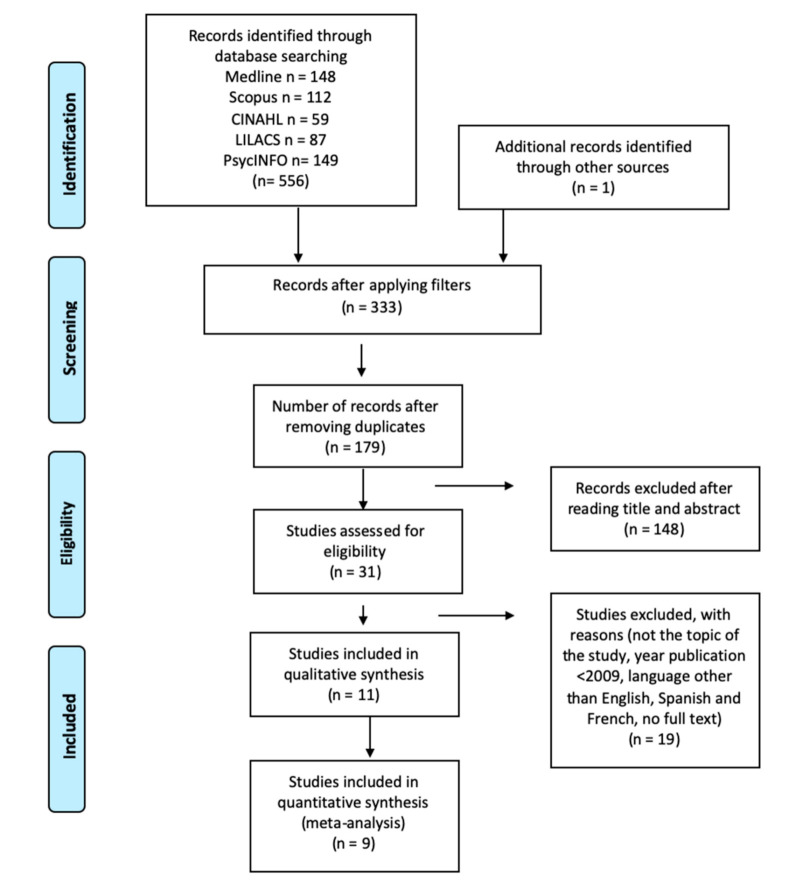
Flow diagram in the selection of articles according to Preferred Reporting Items for Systematic Reviews and Meta-analyses (PRISMA).

A full text reading of these 31 articles was performed, after which a series of articles was deleted according to the inclusion criteria, as can be seen in Figure 1, thus obtaining a final sample of *n* = 11. An analysis of the biases was carried out using the STROBE, CONSORT and CASPE tools (Table S1). For the assessment of the methodological quality of the observational studies, the STROBE statement was used. All studies above 11 of the 22 items were considered valid. CONSORT was used for the evaluation of clinical trials. It was estimated as a cut score of 13/25. CASPE was used for qualitative studies too.

The selection process was assessed by two investigators independently and in case of a discrepancy a third decided. A Kappa indict was obtained from 0.91.

### 2.4. Data Coding

A data coding handbook was used. The following variables were extracted from each study: (a) name of the lead author; (b) year of publication; (c) language of the publication (English–Spanish–French); (d) country where the study was conducted; (e) type of study design; (f) total of the sample of retirees or individuals of retirement age in each study; (g) depression scale used in the study; (h) prevalence of study participants suffering from depression; (i) main results; (j) psychoeducational approach.

The prevalence rates or the number of retirees suffering from depression were obtained directly from each study (total sample of the study and sample with the diagnosis). The inter-researcher reliability of the data coding process was verified by calculating the intraclass correlation coefficient.

### 2.5. Data Analysis

The data analysis was performed by means of the StatsDirect^®^ meta-analysis software package (StatsDirect Ltd., Cambridge, UK). First a sensitivity analysis was conducted to determine if excluding any of the included studies produced significant changes to the results. Apart from that, the publication bias was evaluated by using Egger’s regression test. The prevalence and the confidence intervals were calculated by meta-analyzing random effects. The heterogeneity of the sample was determined by the I^2^ index [18].

## 3. Results

### 3.1. Characteristics of the Study Sample

A total of 11 articles were finally selected, 18.1% (*n* = 2) of which were published in 2018, the same number in 2017, 2015, 2013, and 2009, and 9.1% (*n* = 1) in 2012. The language of the publications was mostly English, with 90.1% (*n* = 10) of the sample in that language, versus one article published in Spanish (8.3%). The countries where the studies were published were mainly the United States, with 27.2% of the sample (*n* = 3), followed by Greece (*n* = 2) and Australia (*n* = 2), both with 18.1%, whereas Singapore, Spain, France and Canada were represented by one with 9.1% (*n* = 1) each.

Regarding the type of design, cross-sectional descriptive cohort studies predominate with 45.4% of the sample (*n* = 5), followed by cohort studies with 27.3% (*n* = 3), nonrandomized clinical trials with 18.8% (*n* = 2) and, finally, a mixed study (quali-quantitative) with 9.1%, as shown in Table S1.

Table 2 shows the descriptive data of the sample of retirees included in each of the studies (gender distribution, mean age of retirement, mean time from retirement and the diagnostic of depression).

### 3.2. Prevalence of Depression

The scales and measurement instruments to evaluate depression most used and collected in the selected articles were mostly the following: “Beck’s Depression Inventory” (BDI-2) with 44.5% (*n* = 5), the “Yesavage’s Geriatric Depression Scale” (GDS) in 18.2% of the sample (*n* = 2), followed by the “Centre for Epidemiological Studies-Depression” (CES-D) scale, the “Glasgow’s Depression” (GDS-G) scale, the “Hospital Anxiety and Depression Scale” (HADS) scale, the “Quality of Life Index” (QLI), or the ”Mini Psychiatric Assessment for Adults with Developmental Disabilities” (Mini PASS-ADD).

According to the results obtained in the studies included in this systematic review, it is concluded that the retirees with the highest prevalence of depression are the ones who retire in a mandatory fashion or due to illness [20,28,29].

According to the data found in this review, the estimated prevalence of depression in retirees ranges from 0.42 to 22.7%, differentiating between mandatory and voluntary retirement, as well as by the level of depression suffered: minimum (46.7–66.7%), slight (10–40%), moderate (17.1–24%) and severe (3.8–16.7%).

### 3.3. Meta-Analysis of the Prevalence of Depression in Retirees

The total sample of retirees collected in the systematic review is *n* = 3019, eventually including a total of *n* = 360 retirees in the meta-analysis, as two articles had to be discarded from the meta-analysis for not contributing data on the prevalence of depression (*n* = 9).

The following table shows the absolute figures of the prevalence of depression in the different samples of retirees from the selected articles (Table 3). The sample taken from the studies is from the retired in general population, which was analyzed by the mentioned scales.

When the sensitivity analysis was performed, the estimated value of the prevalence did not change in a statistically significant way when each of the studies was removed from the analysis. An Egger’s analysis showed that there was no publication bias, with its results showing no statistical significance (*p* = 0.121).

In the heterogeneity analysis, the I^2^ index was 98.7% (95% CI = 98.4–98.9%), showing high heterogeneity.

**Figure 2:**
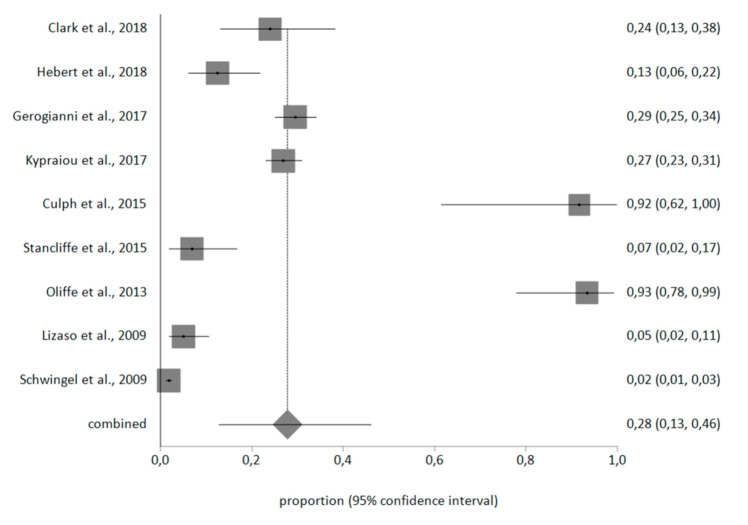
Forest plot of the prevalence of depression in retirees.

Regarding the prevalence values obtained in the meta-analysis, the prevalence of depression in retirees was 28% (95% CI = 13–46%), as can be seen in Figure 2.

## 4. Discussion

After analyzing the 11 articles included in this systematic review, and answering to the two-fold objective of this paper (to analyze the prevalence of depression in retirees and the psychoeducational approach to depression), the results reveal a 28% prevalence of depression in retirees, which shows that almost one-third of this collective population have this condition.

The scarce number of research studies on the theme reveal very dissimilar percentages [30,31] and it is difficult to compare them, as has already been seen in the very articles included in the meta-analysis [19,20,21,22,23,24,26,27]. Even so, meta-analyzing the collected data on the prevalence of this disease gives as a broad view of the magnitude of the problem and allows for the estimation of a percentage which unveils the real importance of the phenomenon, so as to face it with strategies of prevention and early detection [18,19,20].

To this should be added that the majority of the studies included in this review (41.6%) use “Beck’s Depression Inventory”, whose test has a high reliability with a Cronbach’s Alpha of 0.91 [32].

The bottom line is that the results highlight the sanitary role in the psychoeducational approach with 41.6% (*n* = 5), followed by prevention with 25% (*n* = 3) and, finally, detection and adherence to the treatment with 16.6% each (*n* = 2).

Some of the articles included in this review propose the realization by primary care nursing professionals programs aimed at evaluating and assisting people who are in this period of their lives [20,21,22], helping people to find new activities that motivate them [29], encouraging them to participate in community groups [24], helping individuals to deal with the new situation [22] and finding activities that increase their self-esteem again [26].

According to the study carried out by Hebert and collaborators [20], the involvement of the family in this new stage is also important, with nursing being in charge of giving them the information necessary so that they are able to detect in time the appearance of symptoms of depression. A section of retirees heavily affected by depression, according to the study carried out by Lizaso and collaborators [28], are those who retire due to illness, being a field in which nursing could act in order to reduce the prevalence of depression that we find in these people.

In many cases, the transition period towards retirement is loaded with uncertainty among the population, with retirees being more susceptible to developing mental health problems [20]. For this reason, some of the articles included in this review suggest that the health professionals must implement programs intended to evaluate and help the people in this period of their lives [20,21,22], helping individuals in their search for new activities that motivate them [29], to encourage them to participate in community groups [24], to help them build the necessary will power to face the new situation [23], and to find activities that improve their self-esteem [26].

The WHO defends aging as a process in which opportunities are streamlined to have good health, social participation and self-confidence in order to enjoy a good quality of life by means of campaigning for an active aging process [33]. This active aging process would include not only good physical and mental health, but also social participation, thus avoiding treating older people as passive beings and full of needs [5]. Active aging can be applied both individually and in groups, as long as it potentiates the physical, social, and mental well-being of the elderly population; for this reason, both in society and in the socio-sanitary policies, the importance it really has by means of subsidies and promotion initiatives of this movement has to be assigned so that the population are informed. All of the above could help to reduce the great number of deaths produced after exiting the stage which society understands as active, namely the working life [34].

According to a study conducted by Hebert and collaborators [20], the commitment of the family members is also important in this new stage, the multidisciplinary team of health professionals being in charge of giving them the necessary information so that they can promptly detect the onset of the symptoms of depression. In the same line is a study by Wahyuni et al., where the relation between the elderly’s social support and the incidence rate of depression is assessed. The authors conclude that the greater the level of social support, the lower the incidence of depression will be. They stress the importance of health workers, especially nurses, since among their competences is providing health services, especially when they relate to providing social support and to the anticipated incidence of depression in the homes of the elderly [35].

The interchange places for retirees [23,26] are also mentioned, spaces where the individuals can perform activities voluntarily, participate in opinion forums with other retirees, support each other and all supervised and organized by well-trained personnel [36].

A group of retirees which is deeply affected by depression are those who retire due to illness [20,28,29], this being a field in which actions could be taken with the aim of reducing the noticeable prevalence of depression in these individuals. Even so, this study concludes that there is no direct relationship between depression and retirement, with the possibility that retiring increases the levels of depression the person has already suffered from, or that depression is the reason for retiring. In order to elucidate this, further research is needed, using longitudinal studies that allow the establishing of causality relations and verify if aging is a moderator variable in the development of depression in retirees, although there already exist studies with this type of design that have proved depression and retirement are directly related [25].

On the other hand, the different articles included in the review differentiate the prevalence of depression suffered by the participants of each study depending on if its level is minimum, slight, moderate or severe, or moderate, slight or high levels [19,21,22,23,26], the percentage being higher in the case of minimum depression, which coincides and supports the importance of prevention and early detection, areas in which the health professional plays an essential role [37].

### Limitations

Several limitations have occurred, among which the following are worth highlighting: the scarce number of articles found addressing the study theme and the fact that such articles are conducted in various countries with different retirement ages and socioeconomic differences that may influence the retirement process, such as the pensions received, the socio-sanitary system, etc. Another limitation is the fact that part of the sample has had a mandatory retirement by any other disease, which may be a moderating variable to consider. Apart from that, there are few research studies on this theme and the scarce data contributed on the prevalence of depression in retirees are very dissimilar, thus increasing the heterogeneity of the results, as can be verified in the results of the meta-analysis performed in this study. Another influencing factor is the fact that the majority of the studies are cross-sectional and descriptive, further longitudinal research studies being necessary so as to establish causality and determine if aging is a moderator variable in the development of depression in retirees.

## 5. Conclusions

The high percentage of minimum depression is worth highlighting, with the role of health professionals being very important to prevent these levels from advancing into a deeper depression. According to the selected articles in this review, the estimated total prevalence of depression in retirees is 29%. Therefore, and considering the number of retirees worldwide, this implies that one out of three retirees suffers from depression or, more optimistically, one out of four certainly suffers from this disease. Still, a greater diagnostic reliability and appropriate tools are needed to validate this prevalence data, so that we can understand how many retirees meet the diagnostic criteria for major depression.

Answering to the second objective based on the importance of the psychoeducational approach to depression in retirees, it is concluded that the nursing professionals have to be informed to be able to detect the signs and symptoms of depression, especially in primary care, the place where the highest level of autonomy exists and in which a good population screening should be conducted in relation to this disease. The approach of a topic as versatile as depression must be done from a multidisciplinary perspective that integrates all kinds of professionals such as physicians, nurses, psychologists, social workers, occupational therapists, etc.

## Figures and Tables

**Table 1:** Search strategies conducted in the different databases.

Databases	Search Strategy	Filters	Results
Medline	(retirement OR pensions OR unemployment) AND (aged OR aging OR senescence OR elderly) AND (depression OR “depressive symptoms” OR “emotional depression” OR “depressive disorder” OR “depressive syndrome” OR melancholia) AND (nurs* OR “health Personnel”)	(a) Time restriction: 10 years. (b) Languages: English, Spanish and French.	101
Scopus	(retirement OR pensions OR unemployment) AND (aged OR aging OR senescence OR elderly) AND (depression OR “depressive symptoms” OR “emotional depression” OR “depressive disorder” OR “depressive syndrome” OR melancholia) AND (nurs* OR “health Personnel”)	(a) Time restriction: 10 years. (b) Languages: English, Spanish and French	73
CUIDEN	jubilación AND depresión	(a) Time restriction: 10 years. (b) Languages: English, Spanish and French	0
CINHAL	(retirement OR pensions OR unemployment) AND (aged OR aging OR senescence OR elderly) AND (depression OR “depressive symptoms” OR “emotional depression” OR “depressive disorder” OR “depressive syndrome” OR melancholia) AND (nurs* OR “health Personnel”)	(a) Time restriction: 10 years. (b) Languages: English, Spanish and French.	35
LILACS	(retirement OR pensions OR unemployment) AND (aged OR aging OR senescence OR elderly) AND (depression OR “depressive symptoms” OR “emotional depression” OR “depressive disorder” OR “depressive syndrome” OR melancholia) AND (nurs* OR “health Personnel”)	(a) Time restriction: 10 years. (b) Languages: English, Spanish and French.	43
PsycINFO	(retirement OR pensions OR unemployment) AND (aged OR aging OR senescence OR elderly) AND (depression OR “depressive symptoms” OR “emotional depression” OR “depressive disorder” OR “depressive syndrome” OR melancholia) AND (nurs* OR “health Personnel”)	(a) Time restriction: 10 years. (b) Languages: English, Spanish and French.	81

**Table 2:** Prevalence of depression in retirees.

Authors and Year	Gender Distribution	Mean Age of Retirement (Years)	Mean Time from Retirement (Years)	Diagnostic of Depression
Clark et al., 2018 [19]	Female *n* = 2 (4%)	M = 76.8 SD = 7.42 R = 64–88	-------------	Scale = GDS Diagnosed by = Geriatrician
Hebert et al., 2018 [20]	Female *n* = 80 (100%)	Ages 55 and older	-------------	Scale = Ad-hoc Diagnosed by = -----------------
Gerogianni et al., 2017 [21]	Female *n* = 152 (36.7%)	M = 63.54 R = 54–72	M = 1.5	Scale = BDI-II and HADS Diagnosed by = PCP
Kypraiou et al., 2017 [22]	Female *n* = 27 (5.4%)	M = 54.1 SD = 7.4	M = 6.1 SD = 5.6	Scale = BDI-II Diagnosed by = PCP
Culph et al., 2015 [23]	Male *n* = 12 (100%)	M = 67.4 R = 52–77	-------------	Scale = BDI-II Diagnosed by = PCP
Stancliffe et al., 2015 [24]	Female *n* = 16 (29%)	M = 55.6 SD = 6.6 R = 44–72	-------------	Scale = GDS-G and Mini PASS-ADD Diagnosed by = PCP
Zivin et al., 2013 [25]	Female *n* = 4195 (51.4%)	M = 63.8 SD = 8.3	-------------	Scale = CES-D Diagnosed by = ------------------
Oliffe et al., 2013 [26]	Male *n* = 30 (100%)	M = 67.8 R = 55-82	-------------	Scale = BDI-II Diagnosed by = self-identified (*n* = 10); formally diagnosed by a physician (*n* = 20)
Pérès et al., 2012 [27]	Female *n* = 401 (40%)	M = 75.1 SD = 6.6	-------------	Scale = CES-D Diagnosed by = Neuropsychologist Interview
Lizaso et al., 2009 [28]	Female *n* = 37 (31.9%)	M = 66.5 SD = 5.60	*n* = 99 (83.2%) before 65 *n* = 20 (16.8%) after 65	Scale = GADS Diagnosed by = Psychiatrist
Schwingel et al., 2009 [29]	Female *n* = 1159 (66.1%)	M = 66.9 SD = 7.8	-------------	Scale = GDS Diagnosed by = Geriatrician

*Note:* BDI-II = Beck’s Depression Inventory, 2nd Edition; CES-D = Centre for Epidemiological Studies-Depression scale; GADS = Goldberg’s anxiety and depression scale; GDS = Yesavage’s Geriatric Depression Scale (abbreviated); GDS-G = Glasgow’s Depression Scale; HADS = Hospital Anxiety and Depression Scale; M = mean age; Mini PASS-ADD = Mini Psychiatric Assessment for Adults with Developmental Disabilities; *n* = total sample of retirees; PCP = Primary Care Physician; R = range of age; SD = standard deviation.

**Table 3:** Prevalence of depression in retirees.

Authors and Year	*n*_total_ of Retirees	*n*_total_ of Retirees with Depression
Clark et al., 2018 [19]	50	12
Hebert et al., 2018 [20]	80	10
Gerogianni et al., 2017 [21]	414	122
Kypraiou et al., 2017 [22]	502	135
Culph et al., 2015 [23]	12	11
Stancliffe et al., 2015 [24]	58	4
Oliffe et al., 2013 [26]	30	28
Lizaso et al., 2009 [28]	119	6
Schwingel et al., 2009 [29]	1754	32

## Supplementary Materials

The following are available online at https://www.mdpi.com/2227-9032/8/3/321/s1, Table S1: Articles selected in the systematic review.
