# Supplementary material for: Prevalence of Depression in Retirees: A Meta-Analysis
Source: Healthcare (Basel). 2020 Sep 4;8(3):321. doi: 10.3390/healthcare8030321 (PMC7551681; doi:10.3390/healthcare8030321)
Supplement: Supplementary file 1 [file healthcare-08-00321-s001.pdf]

Table S1. Articles selected in the systematic review.

Author(s) and Year Assessment of Biases	Language/ Country	Type of Study Design	Sample (Retirees)	Depression Scale Used	Prevalence of Depression	Most Noticeable Results	Psychoeducational Approach
Clark et al., 2018 [19] STROBE 15/22	English/USA	Cross-sectional and descriptive	n = 50	GDS	−24% depression (N = 12) −40% slight depressive symptoms (N = 20)	- Interdisciplinary care is indispensable to face the multiple comorbidities and complexities of care in geriatric mental health.	The importance stands out of the approach of a multidisciplinary team, as well as when the moment comes for the patient to maintain a good level of adherence to the treatment.
Hebert et al., 2018 [20] STROBE 16/22	English/USA	Cross-sectional and descriptive	n = 80	Ad-hoc	- Mandatory retirement (N = 22) 22.7% suffer from depression (N = 5) -Voluntary retirement (N = 58) 8.6% suffer from depression (N = 5)	- The individuals who retire in a mandatory fashion have a worse mental health than those who retire voluntarily.	The functions of the nursing professionals in retirees approach the assessment and follow-up of the pharmacological treatment and complying with the therapeutic regime, mental health evaluation, and providing educational and therapeutic resources during the transition towards retirement.

Gerogianni et al., 2017 [21] STROBE 19/22	English/Greece	Cross-sectional and descriptive	n = 414	- BDI-II.<br>- HADS	- General depression: 29.4% (N = 122)<br>-Moderate levels: 17.1% (N = 71)<br>-High levels: 12.3% (N = 51)	Women and pension beneficiaries present higher levels of depression. Anticipated retirement is understood as unemployment, being related to psychological problems, loss of self-esteem, anxiety, and depression.	It is suggested to implement educational programs to inform the patients. Additionally, nursing plays an important role when executing own and effective interventions.
Kypraiou et al., 2017 [22] STROBE 19/22	English/Greece	Cross-sectional and descriptive	n = 502	BDI-II	Slight to moderate symptoms: 23.2% (N = 116)<br>Severe depression: 3.8% (N = 19)	- 38.6% of the individuals did not prepare for their retirement.<br>- The individuals who introduced new interests and activities in their daily lives after retiring presented lower levels of depression.<br>- In the individuals who present higher levels of depression after retiring, these levels are related to previous and stressing working conditions and family problems.	A positive guidance is considered fundamental during the crucial period of retirement, since it can help people to maintain control over the depressive symptoms, so that they can focus on the positive aspects of the "big change" inherent to this process.

Culph et al., 2015 [23] STROBE 19/22	English/Australia	Cross-sectional and descriptive	*n* = 12	BDI-II	- Minimum: 66.7% (*N* = 8)<br>- Slight: 16.7% (*N* = 2)<br>- Moderate: 0% (*N* = 0)<br>- Severe: 8.3% (*N* = 1)<br>- N/A: 8.3% (*N* = 1)	- It mentions the importance of staying busy after retirement, searching for activities and hobbies that keep people active.<br>- The participants mentioned a reduction in the number of opportunities to socialize and a greater feeling of isolation.	Nursing is responsible for fostering an appropriate setting for the retirees to feel comfortable and to express their feelings and problems, in order to share life experiences about the retirement process and the depressive symptoms.
Stancliffe et al., 2015 [24] CONSORT 12/25	English/Australia	Not randomized clinical essay	*n* = 58	- GDS-G<br>- Mini PASS-ADD	- Intervention group (n = 28):<br>Pre-test: 7.21% (*N* = 2)<br>Post-test: 6.96% (*N* = 2)<br>- Comparison group (*n* = 28):<br>Pre-test: 5.26% (*N* = 1)<br>Post-test: 5.52% (*N* = 2)	- A limitation of this study is the reduce number of the sample; even so, an improvement can be appreciated in the levels of depression between the pre-test and the post-test.<br>- Participating in community support groups is a good option for the development of an adequate retirement in disabled patients.	Participating in community groups run by the appropriate professionals is a good option to develop a retirement lifestyle in people, which fosters the prevention of depression.

Zivin et al., 2013 [25] STROBE 17/22	English/USA	Cohort longitudin al	*n* = 8,163	CES-D	-------------	This study highlights a key finding: there is a greater probability of suffering from depression at the moment of retiring than if a person continues working. Depression imposes a high cost upon the State. Older adults with depression can negatively affect the children living with them.	Nursing must evaluate people with chronic diseases and make emphasis on the early detection of depression. Apart from that, the availability of psychosocial interventions must be expanded, which could be beneficial for the patients, the caregivers, and the society.
Oliffe et al., 2013 [26] CASPE 9/10	English/Can ada	Qualitative	*n* = 30	BDI-II	- Minimum: 46.7% (*N* = 14)<br>- Slight: 10% (*N* = 3)<br>- Moderate: 20% (*N* = 6)<br>- Severe: 16.7% (*N* = 5)<br>- N/A: 6.6% (*N* = 2)	- When they retired, many participants lost the motivation to get up in the morning.<br>- They perceive retirement as a threat.<br>- Their levels of depression increased when they had difficulties finding activities after retiring.<br>- Some participants linked retirement with suicidal thoughts.	Receiving help from the health professionals who proposed activities like therapy can strengthen self-esteem. This study supports the initiative of homes for retirees as a way of providing socialization opportunities and of having a better transition towards retirement, more progressive and with fewer complications, like loneliness and depression.

Pérès et al., 2012 [27] STROBE 18/22	English/France	Cohort longitudinal	*n* = 1002	CES-D	-------------	- Population aging and the increasing number of chronic diseases and disorders related to age increase the demand for social and health services.	It is important to consider the nutrition habits of the retiree, to evaluate the consumption of alcohol and tobacco, to obtain anthropometric and blood pressure measurements, and to assess muscular strength, among others, to guarantee a better quality of life in the aging process.
Lizaso et al., 2009 [28] CONSORT 13/25	Spanish/ Spain	Not randomized clinical essay	*n* = 119	Goldberg's anxiety and depression scale	According to the type of retirement: - Anticipated: 0.55% (*N* = 1) - Pre-retirement: 1.02% (*N* = 1) - Due to illness: 1.80% (*N* = 2) - Mandatory: 1.19% (*N* = 1) - Voluntary: 0.42% (*N* = 1)	- In this study the main conclusion is that, if the person suffers from depression, the disease can worsen after retiring; but retirement itself does not produce depression. - People who retire due to illness or in a mandatory fashion present higher levels of depression.	In general, retiring due to illness has the worst prognosis, being a risk factor even for psychological well-being; for this reason, the health professionals must be aware of this problem and implement preventive measures aimed at preventing depression.

Schwingel et al., 2009 [29] STROBE 18/22	English/Singapore	Cohort longitudinal	*n* = 1754	GDS	- Not voluntary retirees (*n* = 1360): 1.91% (*N* = 26)<br>- Voluntary retirees (*n* = 193): 1.27% (*N* = 3)<br>- Still working (*n* = 201): 1.39% (*N* = 3)	The individuals who retire voluntarily are the ones that least suffer from depression, followed by the people in retiring age who keep on working, and the non-voluntary retirees present the highest levels of depression. Non-voluntary retirees present higher levels of depression and a worse cognitive state.	Performing activities after retirement can foster a positive adjustment at the psychological and social levels; for this reason, promoting well-being and health must be considered.

Note: BDI-II = Beck's Depression Inventory, 2nd Edition; CASPE = Critical Appraisal Skills Programme; CES-D = Centre for Epidemiological Studies-Depression scale; CONSORT = Consolidated Standards of Reporting Trials; GDS =Yesavage's Geriatric Depression Scale (abbreviated); GDS-G = Glasgow's Depression Scale; HADS = Hospital Anxiety and Depression Scale; QLI = Quality of Life Index; Mini PASS-ADD = Mini Psychiatric Assessment for Adults with Developmental Disabilities; *n* = total sample of retirees; *N* = number of the sample affected by depression; STROBE = Strengthening the Reporting of Observational Studies in Epidemiology.
